# An Evolutionary-Conserved Function of Mammalian Notch Family Members as Cell Adhesion Molecules

**DOI:** 10.1371/journal.pone.0108535

**Published:** 2014-09-25

**Authors:** Akihiko Murata, Miya Yoshino, Mari Hikosaka, Kazuki Okuyama, Lan Zhou, Seiji Sakano, Hideo Yagita, Shin-Ichi Hayashi

**Affiliations:** 1 Division of Immunology, Department of Molecular and Cellular Biology, School of Life Science, Faculty of Medicine, Tottori University, Yonago, Tottori, Japan; 2 Department of Hematology and Oncology, Division of Internal Medicine, Tokai University School of Medicine, Isehara, Kanagawa, Japan; 3 Department of Pathology, Case Western Reserve University, Cleveland, Ohio, United States of America; 4 Corporate R&D Laboratories, Asahi Kasei Corporation, Fuji, Shizuoka, Japan; 5 Department of Immunology, Juntendo University School of Medicine, Bunkyo-ku, Tokyo, Japan; Instituto de Medicina Molecular, Portugal

## Abstract

Notch family members were first identified as cell adhesion molecules by cell aggregation assays in *Drosophila* studies. However, they are generally recognized as signaling molecules, and it was unclear if their adhesion function was restricted to *Drosophila*. We previously demonstrated that a mouse Notch ligand, Delta-like 1 (Dll1) functioned as a cell adhesion molecule. We here investigated whether this adhesion function was conserved in the diversified mammalian Notch ligands consisted of two families, Delta-like (Dll1, Dll3 and Dll4) and Jagged (Jag1 and Jag2). The forced expression of mouse Dll1, Dll4, Jag1, and Jag2, but not Dll3, on stromal cells induced the rapid and enhanced adhesion of cultured mast cells (MCs). This was attributed to the binding of Notch1 and Notch2 on MCs to each Notch ligand on the stromal cells themselves, and not the activation of Notch signaling. Notch receptor-ligand binding strongly supported the tethering of MCs to stromal cells, the first step of cell adhesion. However, the Jag2-mediated adhesion of MCs was weaker and unlike other ligands appeared to require additional factor(s) in addition to the receptor-ligand binding. Taken together, these results demonstrated that the function of cell adhesion was conserved in mammalian as well as *Drosophila* Notch family members. Since Notch receptor-ligand interaction plays important roles in a broad spectrum of biological processes ranging from embryogenesis to disorders, our finding will provide a new perspective on these issues from the aspect of cell adhesion.

## Introduction

Notch receptors and DSL (Delta-Serrate-Lag2) ligands are single pass transmembrane molecules that contain a series of epidermal growth factor (EGF)-like repeats in the extracellular domain (ECD) and are conserved in metazoan species [1], [2]. They are now recognized as one of the core signaling pathways that regulate diverse biological processes ranging from embryogenesis to the maintenance of tissue homeostasis in adults [3]–[7]. Because of its transmembrane nature, activation of the Notch signaling pathway is dependent on direct cell-to-cell contact. Notch receptor-ligand binding between neighboring cells induces the successive proteolytic cleavage of the receptor by a disintegrin and metalloproteases (ADAMs) and γ-secretase complex at the extracellular and transmembrane domain, respectively. This permits the translocation of the intracellular domain into the nucleus, thereby inducing the transcription of Notch target genes such as the Hes (hairy and enhancer of split) and Hey (hairy and enhancer-of-split related with YRPW motif) families [8].

Notch family members were originally identified in the fly *Drosophila*, which has one Notch receptor and two distinct families of DSL ligands; Delta and Serrate, characterized by the presence of a conserved DSL domain [9]. The physiological interaction between the Notch receptor and DSL ligands was first inferred by cell aggregation assays. *Drosophila*-cultured S2 cells over-expressing Notch specifically aggregated with S2 cells over-expressing Delta or Serrate [10], [11]. This process was found to be dependent on specific sequences and Ca^2+^-binding sites within the ECD of these molecules, and was also preserved even when cell metabolism was arrested [10], [11]. A previous study also reported that Notch bound to Delta with a strong adhesion force [12]. These findings indicated that both *Drosophila* DSL ligands exhibited the function of cell adhesion molecules as well as signaling molecules via the Notch receptor. In spite of these early findings, Notch family members have not generally been recognized as cell adhesion molecules, and it remains unclear whether this adhesion function is restricted to *Drosophila*.

Rodents and humans have more diversified Notch family members; four Notch receptors (Notch1–Notch4) and five DSL ligands, classified as two families, Delta-like (Dll1, Dll3 and Dll4) and Jagged (Jag1 and Jag2), based on homology to their *Drosophila* prototypes, Delta and Serrate, respectively [3], [7], [9]. We previously investigated the role of mouse Dll1, the structurally closest relative to Delta among the Delta-like family, in cell adhesion [13]. Using stromal cells enforced to express Dll1 [14] and cultured mast cells (MCs), a hematopoietic cell lineage mainly expressing Notch2, we demonstrated that the adhesion of MCs to Dll1-expressing stromal cells was markedly stronger than that to control stromal cells. The enhanced adhesion of MCs to stromal cells was dependent on Notch receptor(s)-Dll1 binding than to the activation of Notch downstream effectors, which suggested that Dll1 functions as a cell adhesion molecule via Notch receptor(s) [13].

Of the mammalian DSL ligands, Dll1, Dll4, Jag1, and Jag2 are thought to possess a conserved ability to bind and activate any of the four Notch receptors, in spite of their structural differences from *Drosophila* DSL ligands. For example, Dll4 was shown to lack a conserved ECD motif called the DOS (Delta and OSM-11 like) domain, which is known to contribute to receptor binding [5]. Jag2 lacks a conserved intracellular PDZ (PSD-95/Dlg/Zo-1)-ligand motif that mediates interactions with PDZ-containing scaffold/adaptor proteins [5], [15]. Previous studies identified Dll3 as a significantly divergent ligand that lacks the structural features to bind Notch receptors on adjoining cells and, therefore, is not considered as an activating ligand [16]–[18]. Because the signaling function of *Drosophila* DSL ligands is conserved in mammalian Notch ligands, we investigated whether the cell adhesion function of DSL ligands was also conserved among diversified mammalian Notch ligands.

In the present study, we evaluated the function of all mammalian DSL ligands as cell adhesion molecules using an adhesion assay with MCs and stromal cells forced to express each ligand.

## Materials and Methods

### Mice and animal care

C57BL/6J mice (Japan CLEA, Tokyo, Japan) were bred in a specific pathogen-free facility. Experiments were approved and performed in accordance with the guidelines of the Animal Care and Use Committee of Tottori University.

### Bone marrow-derived cultured MCs

Cultured MCs were generated as described [13]. Cells from the femora of C57BL/6J mice (8 to 12-wk-old) were cultured in minimum essential medium alpha (MEMα; Gibco-BRL, Grand Island, NY) supplemented with 10% fetal bovine serum (FBS) (JRH Biosciences, Lenexa, KS), antibiotics (penicillin and streptomycin, Meiji Seika, Tokyo, Japan), and 50 U/ml recombinant mouse interleukin-3 (rmIL-3) (a gift from Dr. Sudo, Toray Industries, Inc., Kanagawa, Japan) at 37°C with 5% CO_2_. Non-adherent cells were placed into fresh media every 5 days. After more than 7 weeks, more than 97% of cells were MCs, as judged by the surface expression of Kit by flow cytometry (Fig. S1).

### Stromal Cells

OP9 stromal cell lines transduced with the coding-sequences for the Dll1, Dll3, Dll4, Jag1, or Jag2 genes (OP9-Dll1, -Dll3, -Dll4, -Jag1 or -Jag2), or with the control Ret10 vector (OP9-Ctrl), as described previously [19]. They were maintained in MEMα supplemented with 20% FBS and antibiotics [20].

### Antibodies (Abs)

Following biotin-conjugated monoclonal Abs (mAbs) were used for flow cytometry; hamster anti-mouse Dll1 (HMD1-5), Dll4 (HMD4-1), Jag1 (HMJ1-29), Jag2 (HMJ2-1), Notch1 (HMN1-12), Notch2 (HMN2-35), Notch3 (HMN3-133), or Notch4 (HMN4-14) [21] and hamster IgG isotype control mAb (eBio299Arm, eBioscience, San Diego, CA); rat anti-mouse Kit (ACK2 and ACK4) [22], PDGFRα (APA5, for control staining for Kit) [23], Dll3 (RMD3-13) or TRAIL (N2B2, for control staining for Dll3) [24]. RMD3-13 (IgG2b) was generated by fusing splenocytes from a mouse Dll3-Fc-immunized SD rat with P3U1 myeloma and screening specific reactivity with OP9-Dll3.

Unlabeled rat anti-mouse IL-7Rα mAb (A7R34) [25], ACK2 [22], HMD1-5 [21], HMD4-1 [21], and hamster anti-mouse CTLA4 mAb (UC10-4F10-11) [26] dissolved in PBS were used in the adhesion assay. Sheep anti-mouse Notch1 (AF5267) or Notch2 (AF5196) polyclonal IgGs (which showed less than 1% cross-reactivity with recombinant mouse Notch2 or Notch1, respectively, according to the manufacturer’s instructions) and sheep control IgG (5-001-A) were purchased from R&D Systems (Minneapolis, MN).

### Reagents

Human IgG1 Fc-fused recombinant human DLL1 (DLL1-Fc), DLL4 (DLL4-Fc), and JAG1 (JAG1-Fc), and Flag-tagged human JAG2 (JAG2-Flag) were as described [27], [28]. Human IgG1 (Sigma, St Louis, MO) was used as a control for recombinant Notch ligands. EGTA, sodium azide (NaN_3_), and DMSO were purchased from Wako Pure Chemical Industries (Osaka, Japan). A γ-secretase inhibitor, *N*-[*N*-(3,5-difluorophenacetyl)-L-alanyl]-S-phenylglycine t-butyl ester (DAPT) was purchased from the Peptide Institute (Osaka, Japan).

### Flow cytometry

Sub-confluent OP9 cells were recovered after being incubated with 10 mM EDTA (Wako) in PBS for 20 min on ice. Hank’s solution (Nissui Pharmaceutical, Tokyo, Japan) containing 2.5% heat-inactivated FBS and 0.02% NaN_3_ was used as a staining buffer. After blocking with 33% rabbit serum (Gibco), cells (<10^6^ cells/test) were stained with biotin-conjugated mAbs (50 µg/ml) for 30 min. After washing, cells were stained with 25 µg/ml of phycoerythrin-labeled streptavidin (SouthernBiotech, Birmingham, AL) (for detection of Dll3) or PerCP-Cy5.5-labeled streptavidin (SouthernBiotech) (for detection of other molecules) for 20 min, and dead cells were then stained with propidium iodide (1.4 µg/ml, Sigma). All processes were done on ice. Cells were analyzed with EPICS XL (Coulter, Palo Alto, CA).

### Adipocyte differentiation assay

The adipocyte assay was performed as previously described [13]. Briefly, OP9-Ctrl cells (1.5×10^4^) were plated in the wells of 48-well flat-bottomed culture plates (Corning Costar, Corning, NY) coated with 10 µg/ml of each recombinant Notch ligand or human IgG1 (120 µl/well, overnight at 4°C) and cultured for 5 days. Adipocytes were stained with Oil Red O solution (Sigma) and the numbers of stained cells in a field of the center of wells were counted under a microscope (magnification; x200). An adipocyte differentiation assay was also performed in 96-well plates and the details were shown in Fig. S3.

### RNA interference

A siRNA against Notch2 (Life Technologies, Carlsbad, CA, Oligo ID; MSS207104) and a siRNA negative control high GC (Life Technologies) (at 500 nM respectively), were each transfected into two separate tubes containing 2.5×10^6^ MCs with Nucleofector II (Lonza, Basel, Switzerland) by Program Y-001 using mouse primary fibroblasts Nucleofector Kit (Lonza) following the manufacturer’s instructions. The transfected MCs were used after culturing for 48 hours in the presence of rmIL-3 (50 U/ml).

### Isolation of MCs cultured on OP9 stromal cells

To assess the expression of Notch target genes, MCs were plated on confluent monolayers of OP9 cells with or without DAPT, and were cultured for 24 hours in a humidified atmosphere with 5% CO_2_ at 37°C. Cells were harvested with 0.1% Trypsin containing 0.5 mM EDTA, and were stained with biotinylated anti-Kit mAb (ACK4) and 10% streptavidin particles plus-DM (BD Biosciences, San Jose, CA). Kit-positive cells were isolated with a magnetic cell sorter (BD IMagnet system, BD Biosciences) according to the manufacturer’s instructions. Flow cytometric analysis revealed that the purity of MCs was more than 99%. Details were shown in Fig. S5.

### RNA analysis

Total cellular RNA was purified using ISOGEN (Nippon Gene, Toyama, Japan) and converted into cDNAs with the PrimeScript RT reagent kit with gDNA Eraser according to the manufacturer’s instructions (Takara Bio Inc., Shiga, Japan). The reverse transcriptase (RT)-PCR was conducted with the 20 µl amplification reaction mixture containing 1x PCR Buffer (Toyobo, Osaka, Japan), 0.2 mM dNTPs (Toyobo), 1.5 mM (for *Notch3*) or 1.0 mM (for other genes) MgCl_2_ (Toyobo), 0.6 U of rTaq DNA polymerase (Toyobo), primers (500 nM each), and cDNA (equivalent to 25 ng of total RNA). The PCR conditions were as follows: 94°C for 3 min for primary; 94°C (60 sec), 60°C (45 sec), 72°C (90 sec) for the following 36 (for Notch receptors) or 23 (for *Gapdh*) cycles. The extension time in the last cycle was 270 s. The primers for detection of *Notch1*–*Notch3*, and *Gapdh* were as previously described [29]. The primers 5′-TGTCATCCTGACCAGAGAGCTT-3′ (forward) and 5′-CGTTGATGTCGCGTTCACAG-3′ (reverse) were used for the detection of *Notch4*.

The quantitative real-time PCR was performed using Light Cycler 480 (Roche, Basel, Switzerland) by a shuttle PCR standard protocol according to the manufacturer’s instructions (Takara), with the 20 µl amplification reaction mixture containing 10 µl of SYBR Premix Ex Taq II (Tli RNaseH Plus) (Takara), primers (400 nM each), and cDNA (equivalent to 50 ng of total RNA). The amplification of a single product was verified by melting curve analysis. Crossing point (CP) values were obtained using Second Derivative Max analysis by the Light Cycler 480 software. Gene expression relative to the expression of *Gapdh* was calculated based on the CP values. The sequences of primers were as follows; *Notch1*, 5′-CTGGACCCCATGGACATC-3′ (forward) and 5′-AGGATGACTGCACACATTGC-3′ (reverse); *Notch2*, 5′-TGCCTGTTTGACAACTTTGAGT-3′ (forward) and 5′-GTGGTCTGCACAGTATTTGTCAT-3′ (reverse); *Gapdh*, 5′-GTCTCCTGCGACTTCAACAG-3′ (forward) and 5′-TCATTGTCATACCAGGAAATGAGC-3′ (reverse). The primers for Notch target genes (*Hes1*, *Hey1* and *Hey2*) were shown in Fig. S5.

### Cell adhesion assay

OP9 cells (1.5×10^4^) suspended in MEMα with 20% FBS were seeded in the wells of 48-well plates and cultured for 2 days to prepare confluent monolayers. After washing OP9 cells with PBS, MCs (1.5×10^5^/200 µl/well) resuspended in MEMα with 10% FBS without rmIL-3 were plated with or without reagents, and incubated for 60 min in a humidified atmosphere with 5% CO_2_ at 37°C unless otherwise indicated. Non-adherent MCs in supernatants were recovered after agitation (low speed, scale 5.5) for 30 sec with MicroMixer E-36 (Taitec, Saitama, Japan) and were counted with a hemocytometer. The percentages of non-adherent MCs relative to the ones initially added in the wells were calculated. In some experiments, adhesion assays were performed in 96-well plates (Corning Costar) with OP9 cells (5.0×10^3^/well) and MCs (5.0×10^4^/50 µl/well) as described above.

To fix stromal cells, confluent monolayers of OP9 cells were fixed with 4% paraformaldehyde (PFA, Nacalai Tesque, Inc. Kyoto, Japan) for 5 min at room temperature. After washing OP9 cells three times with PBS, the adhesion assay was performed.

Photomicrographs of adherent MCs were taken immediately after removing the supernatants using a CCD camera (DS-5Mc, Nikon Corporation, Tokyo, Japan) and Digital Sight DS-L2 imaging controller (Nikon).

The details of the adhesion assay with immobilized JAG1-Fc were shown in Fig. S6.

### Statistics

Data are presented as the mean ± SEM of triplicate cultures. All experiments were performed more than twice with similar results unless otherwise indicated, and representative results were shown. Significance was established at *p*<0.05 by an unpaired two-tailed Student’s *t* test.

## Results

### MCs efficiently adhered to stromal cells enforced to express Dll1, Dll4, Jag1, or Jag2

**Figure 1:**
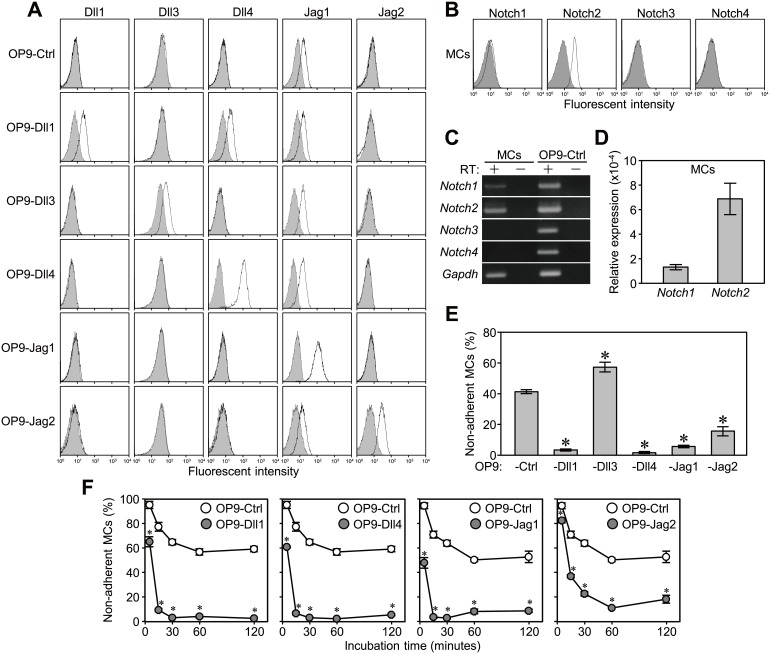
MCs efficiently adhered to OP9-Dll1, -Dll4, -Jag1, and -Jag2, but not OP9-Dll3, than to OP9-Ctrl. (A and B) Flow cytometric analysis of the expression of (A) Dll1, Dll3, Dll4, Jag1, and Jag2 on OP9 stromal cells transduced with each Notch ligand gene, and (B) Notch receptors on MCs after staining with specific mAbs (open histograms) or isotype-matched control mAbs (filled histograms). (C) Total RNA was analyzed by RT-PCR for the expression of Notch receptors in MCs and OP9-Ctrl cells. (D) Relative expression levels of *Notch1* and *Notch2* to *Gapdh* in MCs were analyzed by quantitative RT-PCR. Data represent the mean ± SEM of 3 independent experiments. (E and F) An adhesion assay for MCs on each OP9 cell (E) in a 48-well plate for 60 min and (F) in 96-well plates with serial incubation times of 5, 15, 30, 60, and 120 min. Data represent the percentages of non-adherent MCs (mean ± SEM of triplicate cultures) (*p<0.05 significantly different from OP9-Ctrl at each time point, the Student’s *t-*test).

To clarify the contribution of DSL ligands to cell adhesion, we employed OP9 stromal cells transduced with each ligand gene or control vector (OP9-Dll1, -Dll3, -Dll4, -Jag1, -Jag2 or -Ctrl) [19]. Each transduced Notch ligand was expressed on the cell surfaces (Fig. 1A). All OP9 transductants endogenously expressed Jag1 (Fig. 1A). We employed mouse bone marrow-derived cultured MCs (Fig. S1), predominantly expressing Notch2 on the cell surface, as an indicator of cell adhesion (Fig. 1B). The surface expression level of Notch1 on MCs was very low (Fig. 1B), whereas the expression of *Notch1* mRNA was clearly detected by RT-PCR (Fig. 1C). The expression level of *Notch1* transcript was about one fifth that of *Notch2* transcript (Fig. 1D). The expression of Notch3 and Notch4 in MCs was not detected both by flow cytometry and RT-PCR (Fig. 1B, 1C).

We evaluated the adhesion efficiency of MCs to the confluent monolayers of each OP9 cell by comparing the percentages of floating MCs after a 60-min co-cultivation. MCs did not die or proliferate during the adhesion assay (Fig. S2). There were fewer non-adherent MCs on OP9-Dll1, -Dll4, -Jag1, and -Jag2 than on OP9-Ctrl (Fig. 1E). The percentage of non-adherent MCs on OP9-Dll3 was similar or sometimes higher than that on OP9-Ctrl (Fig. 1E). Time course analysis (5, 15, 30, 60, and 120 min) revealed that the percentage of non-adherent MCs on OP9-Ctrl gradually decreased and plateaued after 60 min (Fig. 1F, open circles). In contrast, the percentage of non-adherent MCs at every time point was significantly lower on OP9-Dll1, -Dll4, -Jag1, and -Jag2 than on OP9-Ctrl (Fig. 1F, filled circles). Furthermore, the adhesion of MCs on OP9-Dll1, -Dll4, and -Jag1 plateaued within 15–30 min, which was markedly earlier than the MCs on OP9-Ctrl.

These results indicated that the increased expression of Dll1, Dll4, Jag1, and Jag2, but not Dll3 on stromal cells induced the enhanced and rapid adhesion of MCs to stromal cells.

### Enhanced adhesion of MCs depended on Notch receptor-ligand binding

**Figure 2:**
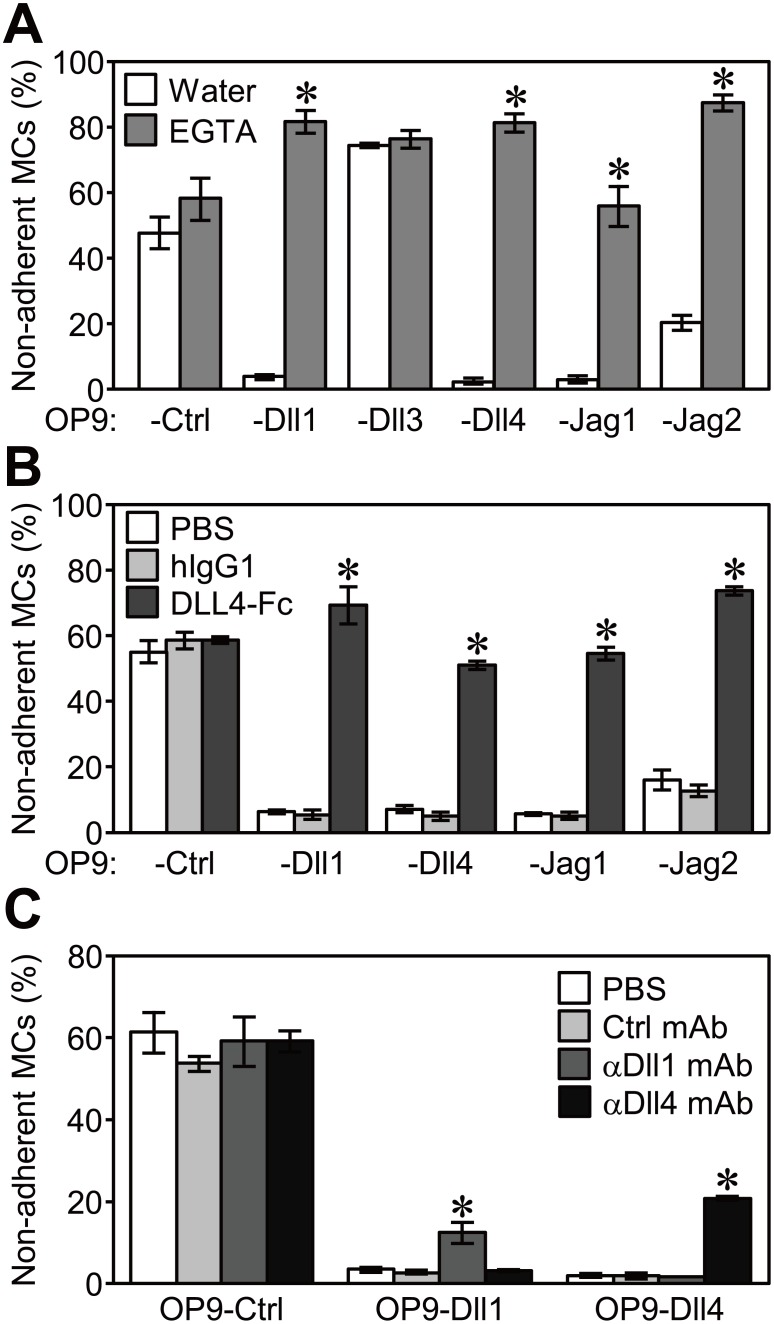
Enhanced MC adhesion to OP9-Dll1, -Dll4, -Jag1, or -Jag2 depended on Notch receptor-ligand interactions. An adhesion assay (60 min) for MCs on each OP9 cell (A) with EGTA (3.0 mM) or the same volume of distilled water (DW, control, 0.6% vol/vol) in a 48-well plate, (B) with 25 µg/ml of soluble recombinant DLL4-Fc, human IgG1 (control), or the same volume of PBS (19.2% vol/vol) in a 96-well plate, (C) with 50 µg/ml of anti-Dll1 mAb, anti-CTLA4 mAb (control), or the same volume of PBS (5.0% vol/vol) in a 96-well plate, (D) with 200 µg/ml of anti-Dll4 mAb, anti-CTLA4 mAb (control), or the same volume of PBS (20.0% vol/vol) in a 96-well plate. Data represent the percentages of non-adherent MCs (mean ± SEM of triplicate cultures) (*p<0.05 significantly different from each control treatment on the same OP9 cells, the Student’s *t-*test).

We determined whether the enhanced adhesion of MCs to OP9-Dll1, -Dll4, -Jag1, or -Jag2 was due to Notch receptor(s)-ligand interactions. Notch receptors have many Ca^2+^ binding sites in their EGF-like repeats, some of which are critical for ligand binding [10], [30]–[32]. Consistent with this finding, the enhanced adhesion of MCs on OP9-Dll1, -Dll4, -Jag1, and -Jag2 was blocked in the presence of EGTA, a selective chelating agent for Ca^2+^ (Fig. 2A). The enhanced adhesion of MCs was also blocked by competitive inhibition with soluble DLL4-Fc (Fig. 2B). Furthermore, the adhesion of MCs to OP9-Dll1 or OP9-Dll4 was significantly inhibited by the addition of antagonistic mAbs against Dll1 (HMD1-5) or Dll4 (HMD4-1), respectively, which were used at concentrations that induced a maximal inhibitory response (Fig. 2C, 2D and Fig. S4) [21]. These results suggested that triggering of the enhanced adhesion of MCs on OP9-Dll1, -Dll4, -Jag1, or -Jag2 was dependent on Notch receptor(s)-ligand interactions. The addition of EGTA or DLL4-Fc had no effect on the adhesion of MCs to OP9-Ctrl, suggesting that endogenously expressed Jag1 on OP9 cells did not markedly contribute to adhesion (Fig. 2A, 2B).

**Figure 3:**
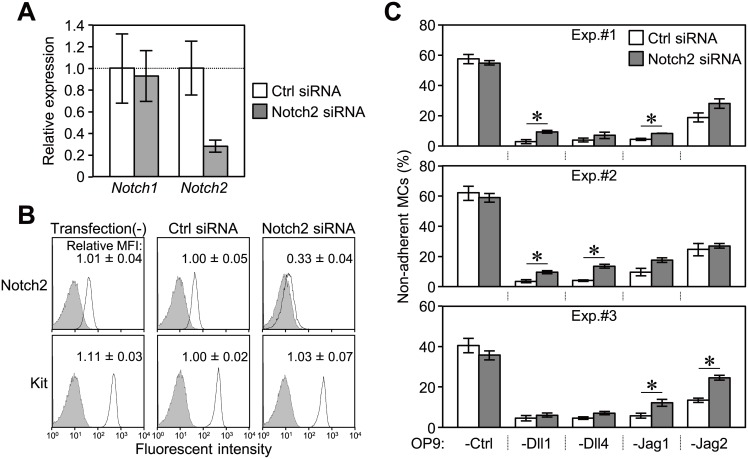
Reduction in Notch2 on MCs did not have marked effects on the enhanced adhesion. MCs were analyzed 48 hours after transfection with siRNA against Notch2 or control siRNA. (A) Relative expression levels of *Notch1* and *Notch2* to *Gapdh* in MCs transfected with each siRNA were analyzed by quantitative RT-PCR. Data represent the mean ± SEM of three independent experiments. (*p<0.05 significantly different from the control siRNA treatment, the Student’s *t-*test) (B) Flow cytometric analysis of the expression of Notch2 and Kit on MCs transfected with each siRNA after staining with specific mAbs (open histograms) or isotype-matched control mAbs (filled histograms). Representative histograms from one of three independent experiments are shown. Numbers indicate the relative mean fluorescence intensities (MFIs) of specific mAbs relative to that of the control siRNA treatment (MFIs of specific mAbs were normalized by the MFIs of control mAbs) (mean ± SEM of three independent experiments). (C) An adhesion assay (60 min) for MCs transfected with each siRNA on each OP9 cell in a 96-well plate. Data represent the percentages of non-adherent MCs (mean ± SEM of triplicate cultures) (*p<0.05, the Student’s *t-*test).

To identify which Notch receptor functioned in the enhanced adhesion of MCs as a counter-receptor for each Notch ligand, we assessed the effects of reducing Notch2 by RNA interference on MC adhesion. The expression of *Notch2* mRNA in MCs was significantly reduced 48-hours after transfection with a siRNA against Notch2 (Fig. 3A), and the surface level of Notch2 was decreased to 33% of siRNA transfected control (Fig. 3B). Transfection with those siRNAs did not influence the expression of Notch1 mRNA (Fig. 3A) and the surface expression of Kit (Fig. 3B). Although not in all experiments, the reduction in Notch2 significantly inhibited the adhesion of MCs on OP9-Dll1, -Dll4, -Jag1, or -Jag2 in three independent assays, MCs still markedly adhered to those stromal cells compared to the response on OP9-Ctrl (Fig. 3C).

**Figure 4:**
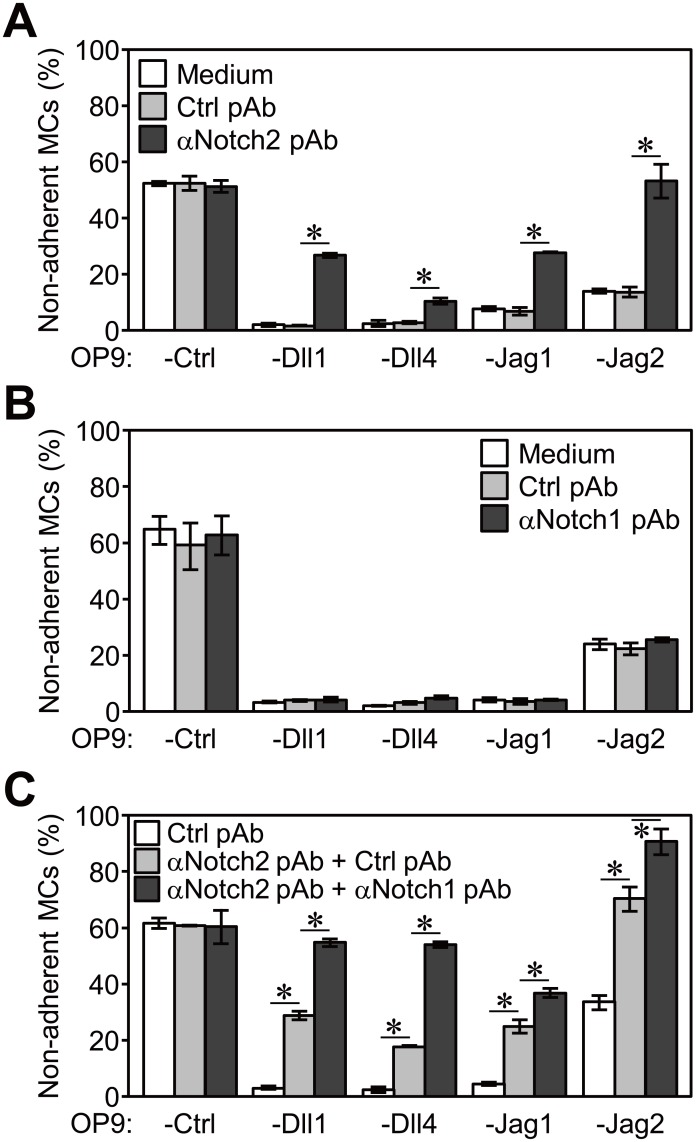
Enhanced adhesion of MCs by Notch ligands involved both Notch1 and Notch2 on MCs. An adhesion assay (60 min) for MCs on each OP9 cell in a 96-well plate (A and B) with or without 10 µg/ml of the indicated polyclonal Ab (pAb) and (C) with control pAb (20 µg/ml), anti-Notch2 pAb (10 µg/ml) plus control pAb (10 µg/ml) or anti-Notch2 pAb (10 µg/ml) plus anti-Notch1 pAb (10 µg/ml). Data represent the percentages of non-adherent MCs (mean ± SEM of triplicate cultures) (*p<0.05, the Student’s *t-*test). Cultures with pAbs contained (A and B) 1.0% PBS (vol/vol) and 38.5 µM NaN_3_ and (C) 2.0% PBS (vol/vol) and 76.9 µM NaN_3_, which had no effect on the adhesion of MCs.

Therefore, we assessed competitive inhibition with an anti-Notch1 or anti-Notch2 polyclonal Ab (pAb). The addition of an anti-Notch2 pAb significantly inhibited the enhanced adhesion of MCs to OP9-Dll1, -Dll4, -Jag1, or -Jag2 (Fig. 4A). In comparison, the addition of an anti-Notch1 pAb had no effect on the adhesion of MCs (Fig. 4B). However, the combined addition of both antibodies more effectively inhibited the adhesion of MCs to OP9-Dll1, -Dll4, -Jag1, and -Jag2 than the addition of an anti-Notch2 pAb alone (Fig. 4C). These results indicated that Notch1 and Notch2 on MCs cooperatively function as receptors for each Notch ligand, directing the marked adhesion of MCs.

### Notch signaling did not account for the enhanced adhesion of MCs

**Figure 5:**
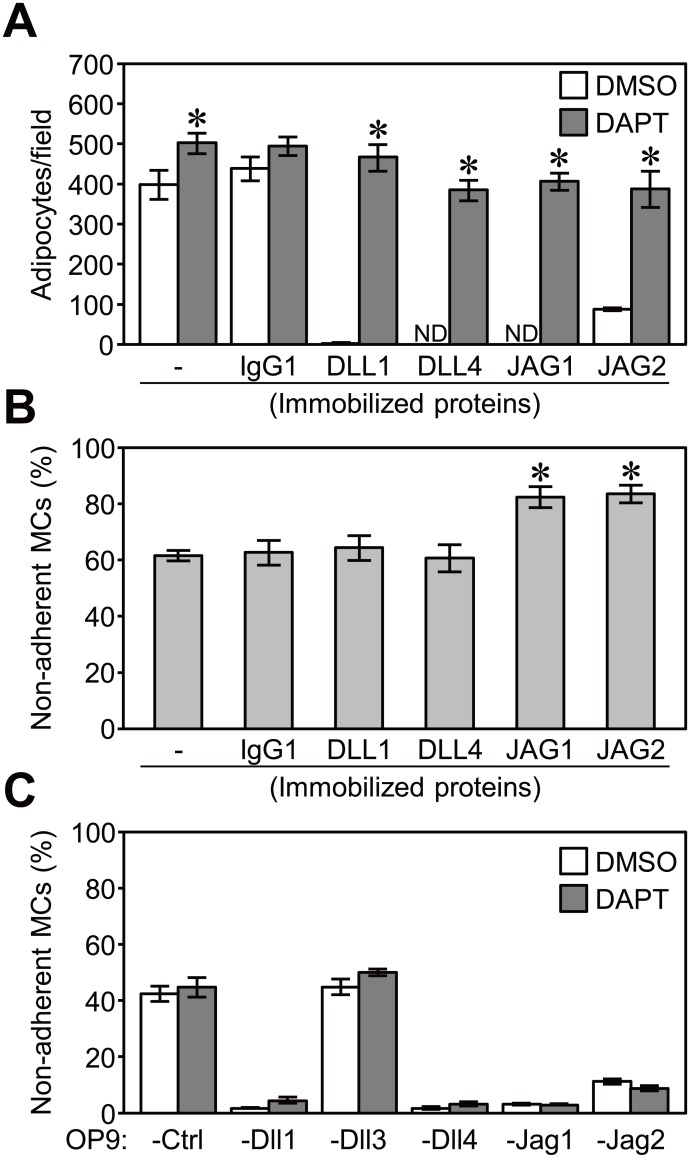
Notch signaling in stromal cells or MCs did not account for the enhanced adhesion. (A) An adipocyte differentiation assay of OP9-Ctrl cells stimulated with immobilized Notch ligands or human IgG1 (control) for 5 days in the presence of DAPT (10 µM) or the same volume of DMSO (control, 0.1% vol/vol). Data represent the numbers of adipocytes in a field (magnification; x200) in the center of the wells (mean ± SEM of triplicate cultures) (*p<0.05 significantly different from IgG1, the Student’s *t-*test). ND: not detected. (B) OP9-Ctrl cells were stimulated with each immobilized Notch ligand or human IgG1 (control) for 2 days in a 48-well plate, and an adhesion assay (60 min) for MCs was then performed. (C) An adhesion assay (60 min) for MCs on each OP9 cell with DAPT (10 µM) or the same volume of DMSO (control, 0.1% vol/vol). (B and C) Data represent the percentages of non-adherent MCs (mean ± SEM of triplicate cultures). (B) *p<0.05 significantly different from IgG1 and (C) no significant differences between the responses with DMSO and DAPT on the same OP9 cells (the Student’s *t-*test).

The enhanced adhesion of MCs may be a consequence of the additional expression of cell adhesion molecule(s) on stromal cells or MCs by Notch signaling. We first examined whether the Notch activation of stromal cells contributed to the enhanced adhesion of MCs because OP9 cells expressed Notch receptors (Fig. 1C). We tested if the treatment of OP9-Ctrl cells with immobilized recombinant Notch ligands could markedly enhance the adhesion of MCs. The activation of Notch signaling in OP9-Ctrl cells was confirmed by an adipocyte differentiation assay [13]. A stimulation with each Notch ligand inhibited the differentiation of OP9-Ctrl cells into adipocytes and this inhibition was reversed by the addition of DAPT (10 µM), an inhibitor of γ-secretase that is essential for Notch signaling (Fig. 5A). The adipocyte differentiation of OP9-Dll1, -Dll4, -Jag1, and -Jag2 was also impaired, while the addition of DAPT during the culture significantly increased adipocyte differentiation (Fig. S3).

We evaluated the adhesion of MCs to OP9-Ctrl cells stimulated for 2 days by immobilized Notch ligands. The stimulation of OP9-Ctrl cells with DLL1-Fc or DLL4-Fc had no effect on the adhesion of MCs (Fig. 5B), with one exception that the DLL4-Fc stimulation increased the adhesion of MCs in one of four independent experiments (non-adherent MCs; 40.2±3.6% [control] vs 26.4±1.5% [DLL4-Fc] [*p*<0.05]). The stimulation of OP9-Ctrl cells with JAG1-Fc or JAG2-Flag had no effect or inhibited the adhesion of MCs in three (JAG1-Fc) or two (JAG2-Flag) of four independent experiments (Fig. 5B). These results suggested that Notch signaling in OP9 stromal cells was not responsible for the enhanced adhesion of MCs.

We next assessed the contribution of Notch signaling in MCs. The transcript levels of the Notch target genes (*Hes1* and *Hey1*) in MCs co-cultured with OP9-Dll1 for 24 hours were significantly higher than those co-cultured with OP9-Ctrl (Fig. S5). The up-regulation of *Hes1* and *Hey1* was significantly inhibited in the presence of DAPT (10 µM) (Fig. S5), suggesting that Notch signaling may have been activated in MCs during the adhesion assay. The addition of DAPT (10, 30, and 100 µM) during the adhesion assay had no effect on the adhesion of MCs to each OP9 transductant (Fig. 5C and data not shown), which suggested that Notch signaling in MCs was also not responsible for the triggering of enhanced adhesion.

### Notch receptor-ligand interactions induced tethering of MCs to stromal cells

**Figure 6:**
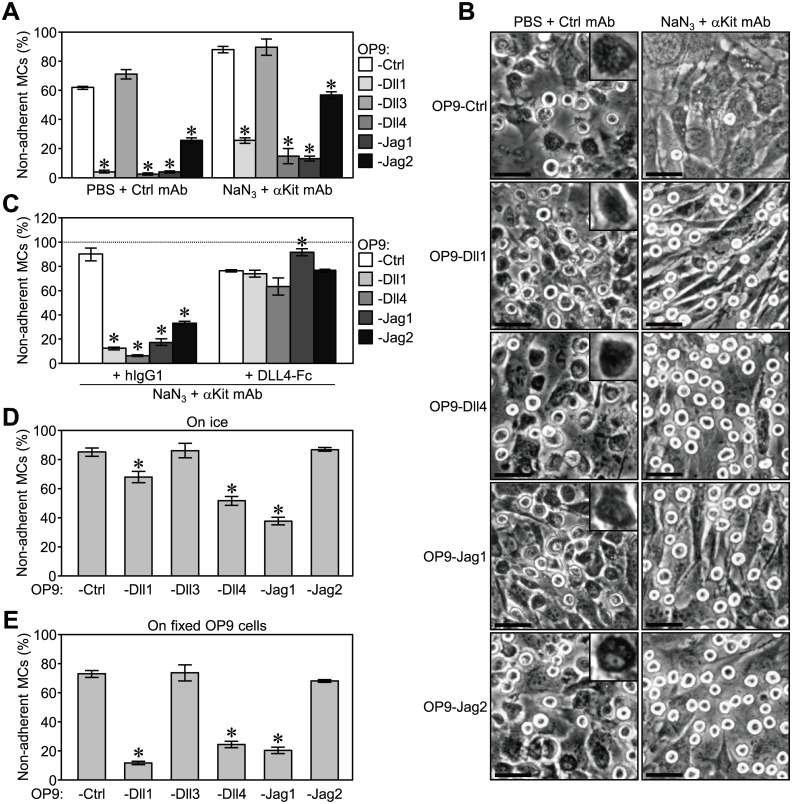
Notch receptor-ligand binding strongly supported the tethering of MCs to stromal cells. (A and B) An adhesion assay with an anti-IL-7Rα mAb (control) or anti-Kit mAb (5 µg/ml each) in the presence of NaN_3_ (50 mM) or the same volume of PBS (0.083% vol/vol) in a 96-well plate. (A) Data represent the percentages of non-adherent MCs (mean ± SEM of triplicate cultures) (*p<0.05 significantly different from OP9-Ctrl with each treatment, the Student’s *t-*test). (B) Representative photomicrographs of adherent MCs on each OP9 stromal cell after the removal of floating cells were shown (original magnification x200). Scale bars; 50 µm. Insets; higher magnification of a spreading adherent MC. (C) An adhesion assay with human IgG1 (control) or DLL4-Fc (25 µg/ml each) in the presence of anti-Kit mAb (5 µg/ml) and NaN_3_ (50 mM) in a 96-well plate. (D and E) An adhesion assay (60 min) in a 48-well plate (D) at 37°C or on ice, and (E) on non-fixed or 4% PFA-fixed stromal cells. (C to E) Data represent the percentages of non-adherent MCs (mean ± SEM of triplicate cultures) (*p<0.05 significantly different from OP9-Ctrl with each treatment, the Student’s *t-*test). In (A), (D) and (E), data displayed significant differences between treatments on the same OP9 cells in most cases (the Student’s *t-*test).

We conducted an adhesion assay under the arrest of cellular metabolism in order to further confirm that the Notch receptor-ligand interaction itself triggered the adhesion of MCs. We previously demonstrated that an anti-Kit mAb combined with NaN_3_, an inhibitor of mitochondrial F-ATPase [33], disrupted the adhesion of MCs on control stromal cells [13]. The adhesion of MCs to OP9-Ctrl and -Dll3 was mostly inhibited in the presence of the above reagents (Fig. 6A). In contrast, although the adhesion of MCs to OP9-Dll1, -Dll4, -Jag1, or -Jag2 was significantly inhibited by this treatment, a large number of MCs still adhered to these stromal cells (Fig. 6A).

MCs in the original suspension culture were spherical in shape and appeared to be refractile under phase-contrast microscopy. While some adherent MCs maintained their original morphology after a 60-min co-culture with stromal cells, some adherent MCs spread on stromal cells with deformed shapes and appeared dark, which may represented a more advanced stage of cell adhesion accompanied by cytoskeletal reorganization (Fig. 6B, left panels) [34], [35]. Most of the adherent MCs on OP9-Dll1, -Dll4, -Jag1, and -Jag2 in the presence of anti-Kit mAb and NaN_3_ maintained a spherical shape and were refractile, which suggested that adherent MCs were tethering in this condition (Fig. 6B, right panels). This tethering of MCs was again blocked by competitive inhibition with soluble DLL4-Fc (Fig. 6C). These results indicated that Notch receptor-ligand binding itself induced the effective tethering of MCs, the first step of cell adhesion.

We performed the adhesion assay on ice to more widely inhibit cellular metabolism. While the adhesion of MCs to OP9-Ctrl was mostly inhibited, significantly large numbers of MCs were still tethered to OP9-Dll1, -Dll4, and -Jag1 on ice (Fig. 6D). In contrast, the enhanced adhesion of MCs to OP9-Jag2 was completely disrupted on ice (Fig. 6D). The enhanced adhesion of MCs on OP9-Jag2 was also suppressed when we performed the adhesion assay on PFA-fixed OP9 stromal cells, while that on OP9-Dll1, -Dll4, and -Jag1 still remained (Fig. 6E). These results indicated that other mechanism(s) in addition to the Notch receptor-ligand interaction may be employed by Jag2 to function in the adhesion of MCs.

We finally determined whether the immobilized Notch ligands on the plastic surface could induce the adhesion of MCs. MCs did not adhere to immobilized JAG1-Fc (10 and 50 µg/ml) in the presence or absence of stem cell factor, which induced the adhesion of MCs to immobilized fibronectin (Fig. S6) [36], [37]. This result indicated that Notch ligands have to reside on cell surfaces to induce the adhesion of MCs.

Taken together, our results suggest that Dll4, Jag1 and Jag2, in addition to Dll1, on stromal cells function as cell adhesion molecules via Notch1 and Notch2 on MCs.

## Discussion

The Notch receptor and its ligands were originally discovered as cell adhesion molecules that induced aggregation in *Drosophila* cultured cells. We here provided evidence that mammalian Notch family members also possess the function of cell adhesion molecules. The increased expression of the murine DSL Notch ligands, Dll1, Dll4, Jag1, and Jag2, but not Dll3, on stromal cells effectively promoted the adhesion of MCs in a Notch receptor-ligand binding-dependent manner. The triggering of enhanced MC adhesion was found to be independent of the activation of Notch signaling in both MCs and stromal cells. In addition, the Notch receptor-ligand binding-dependent tethering of MCs to OP9-Dll1, -Dll4, -Jag1, and -Jag2 occurred even when cell metabolism was arrested (Fig. 6), which further supported Notch receptors and the ligands themselves, rather than the activation of Notch downstream effectors, mediating the effective adhesion of MCs. Thus, the function of cell adhesion molecules is conserved among mammalian as well as *Drosophila* Notch family members.

While the function of adhesion is preserved in all DSL Notch ligands, except for Dll3, the promotion of MC adhesion mediated by Jag2 was weaker and required more time to maximize adhesion of MCs than that mediated by Dll1, Dll4, and Jag1. Unlike other ligand-mediated adhesions, MC adhesion mediated by Jag2 was also disrupted in cultures on ice or on fixed stromal cells. The inability of stromal cells to respond to signal transduction after interactions with MCs was common in the above adhesion assays, suggesting that some additional signaling in OP9-Jag2 cells may be required for Jag2 to function as an adhesion molecule. A previous study reported a difference in the binding ability of Jag2 to Notch2 from the other ligands, and suggested that molecule(s) presented on the cell surface may be required to support the Notch2-Jag2 interaction [38]. Although the underlying mechanism remains unclear, these findings suggest the unique characteristics of Jag2 among the Notch ligands involved in cell adhesion. Unlike other ligands, Dll3 on stromal cells did not promote the adhesion of MCs. Dll3 is a significantly divergent Delta homologue that only shares 36% amino acid sequence homology with Dll1 and lacks the structural features required by other Notch ligands to bind and activate Notch receptors [16]. The majority of Dll3 has been suggested to reside in the Golgi apparatus under physiological conditions and its cell surface expression is likely to only be detected when it is overexpressed [18]. Even under that condition, Dll3 did not activate any of the four Notch receptors on neighboring cells [17], [18]. Therefore, the inability of Dll3 on stromal cells to enhance the adhesion of MCs may reflect its inability to bind to Notch receptors on MCs. Immobilized JAG1-Fc on the plastic surface failed to induce the adhesion of MCs (Fig. S6). This result suggests that Notch ligands may need to reside on cell surfaces or require other molecule(s) on cell membranes to function as cell adhesion molecules.

OP9 stromal cells endogenously expressed Jag1, but did not appear to be directly involved in the adhesion of MCs to OP9-Ctrl, which suggests that Notch ligands may require a certain threshold level of surface expression for their adhesion function. The differentiation of OP9-Ctrl cells into adipocytes, which was inhibited by Notch signaling, was increased when cells were treated with a Notch signaling inhibitor (Fig. 5A and Fig. S3), which suggested that endogenously expressed Jag1 activated Notch signaling among OP9-Ctrl cells. Therefore, the signaling and adhesion functions of Notch ligands may require different expression level thresholds while the latter requires a higher threshold.

We showed the involvement of Notch1 and Notch2 on MCs in the enhanced MC adhesion by Notch ligands. It has yet to be confirmed whether Notch3 and Notch4 also function in cell adhesion. The involvement of Notch1 was unexpected because it was barely detectable on the cell surface of MCs. The inhibition of Notch2 by pAb solely and significantly inhibited enhanced adhesion, whereas the involvement of Notch1 was only observed when Notch2 was inhibited together, which suggested that Notch2 was sufficient for enhanced adhesion. In contrast, reduction in Notch2 on MCs by RNA interference did not have marked effects on the enhanced adhesion of MCs by Dll1, Dll4, Jag1, or Jag2. These results suggested that Notch receptors may only require a low level of expression to induce enhanced cell adhesion, unlike Notch ligands, which may require a high level of expression.

In addition to the aggregation assays performed in early *Drosophila* studies, the findings of pioneering studies have suggested that vertebrate Notch family members also function in cell adhesion. For example, the mouse pro-B cell line, Ba/F3, which hardly adhere to the Chinese hamster ovary (CHO) cell line, can adhere to CHO cells over-expressing mouse Dll1, Jag1, or Jag2 [38], [39]. The over-expression of zebrafish DeltaD or mouse Dll1 in cultured human keratinocytes has been shown to promote their cohesiveness [40]. However, it was not shown whether enhanced adhesion or cohesion resulted from Notch receptor-ligand binding itself or as a consequence of the activation of Notch signaling.

The reason why Notch family members have not generally been recognized as cell adhesion molecules in spite of the findings of these early studies can be attributed to the activation mechanism of the Notch signaling pathway. Notch receptors have to be cleaved by proteases at the ECD in order for subsequent signaling events to be activated. This cleavage was shown to occur immediately after ligand binding [12], [38]. The size of cell aggregates between *Drosophila* S2 cells expressing Notch and those expressing Delta increased in the first 10 min and then decreased [12]. The Ba/F3 cells that adhered to CHO cells expressing Dll1 mostly detached within 2 hours [39]. These findings suggest that cell-to-cell adhesion mediated by Notch receptor-ligand interactions occurs transiently at the very beginning of activation of the Notch signaling pathway. Therefore, it may not be considered as an important cell adhesion mechanism. However, we challenge this view because the enhanced adhesion of MCs mediated by Notch receptor-ligand interactions was not transient in the present study. One possible explanation for this inconsistency is the use of single mutually non-interacting cells (S2 cells) or xenogeneic cells (Ba/F3 and CHO cells) that may have mismatches of the molecules involved in cell adhesion due to species difference. Our results suggest that Notch receptor-ligand interactions can support cell adhesion between normally interacting cells (MCs and stromal cells) for a relatively long time period. This raises the question as to why Notch receptors, which are going to be cleaved eventually, should support cell adhesion. Elucidating the relationship between adhesion and the signaling function of Notch will provide insights into the regulation of these seemingly incompatible functions.

Compared with the information available on their signaling function, little is known about the physiological roles of the cell adhesion function of Notch, even in *Drosophila*. In the mammalian immune system, cell adhesion molecules play pivotal roles in the recruitment of immune cells from the circulation, as well as their retention and localization in normal and inflammatory tissues. Of note, the localization of MCs in the small intestine has been shown to involve Notch2; Notch2-null MCs cannot localize to the epithelium in which Jag1 is abundantly expressed; therefore, they abnormally accumulate in the lamina propria [41]. In addition, evidence to suggest that various chronic inflammatory disorders are accompanied by the aberrantly increased expression of Notch ligands on endothelial cells and stromal cells in inflamed tissues is mounting [42]–[50]. Blocking Notch receptor-ligand interactions has also been suggested to inhibit the accumulation of immune cells such as CD4^+^ T cells and macrophages in inflamed tissues [49], [51], [52]. Therefore, evaluating the role of Notch as an adhesion molecule in light of our findings will provide important insights into the regulation of immune cell dynamics.

Finally, Notch family members are critical to a broad spectrum of biological processes ranging from normal development to maintaining homeostasis in diverse metazoans. The appreciation that Notch family members function as cell adhesion molecules both in fruit fly (arthropod) and mammals (chordate), which diverged from a common ancestor (Urbilateria) in the late Precambrian [53], will give a new perspective on understanding these important issues.

## Supporting Information

Figure S1 **Profiles of MCs used in this study.**
(EPS)

Figure S2 **Survival and proliferation of MCs during the adhesion assay.**
(EPS)

Figure S3 **Adipocyte differentiation of OP9-Ctrl, -Dll1, -Dll4, -Jag1, and -Jag2.**
(EPS)

Figure S4 **Effects of anti-Dll1 and -Dll4 mAbs on the adhesion of MCs at various concentrations.**
(EPS)

Figure S5 **Expression of Notch target genes in MCs cultured on OP9-Ctrl and OP9-Dll1.**
(EPS)

Figure S6 **Adhesion of MCs to immobilized JAG1-Fc.**
(EPS)
