# Correction: An Evolutionary-Conserved Function of Mammalian Notch Family Members as Cell Adhesion Molecules

**DOI:** 10.1371/journal.pone.0115811

**Published:** 2014-12-12

**Authors:** The PLOS ONE Staff

Figures 1, 2, and 6 are incorrect. Please view the corrected Figures 1, 2, and 6 here.

**Figure 1:**
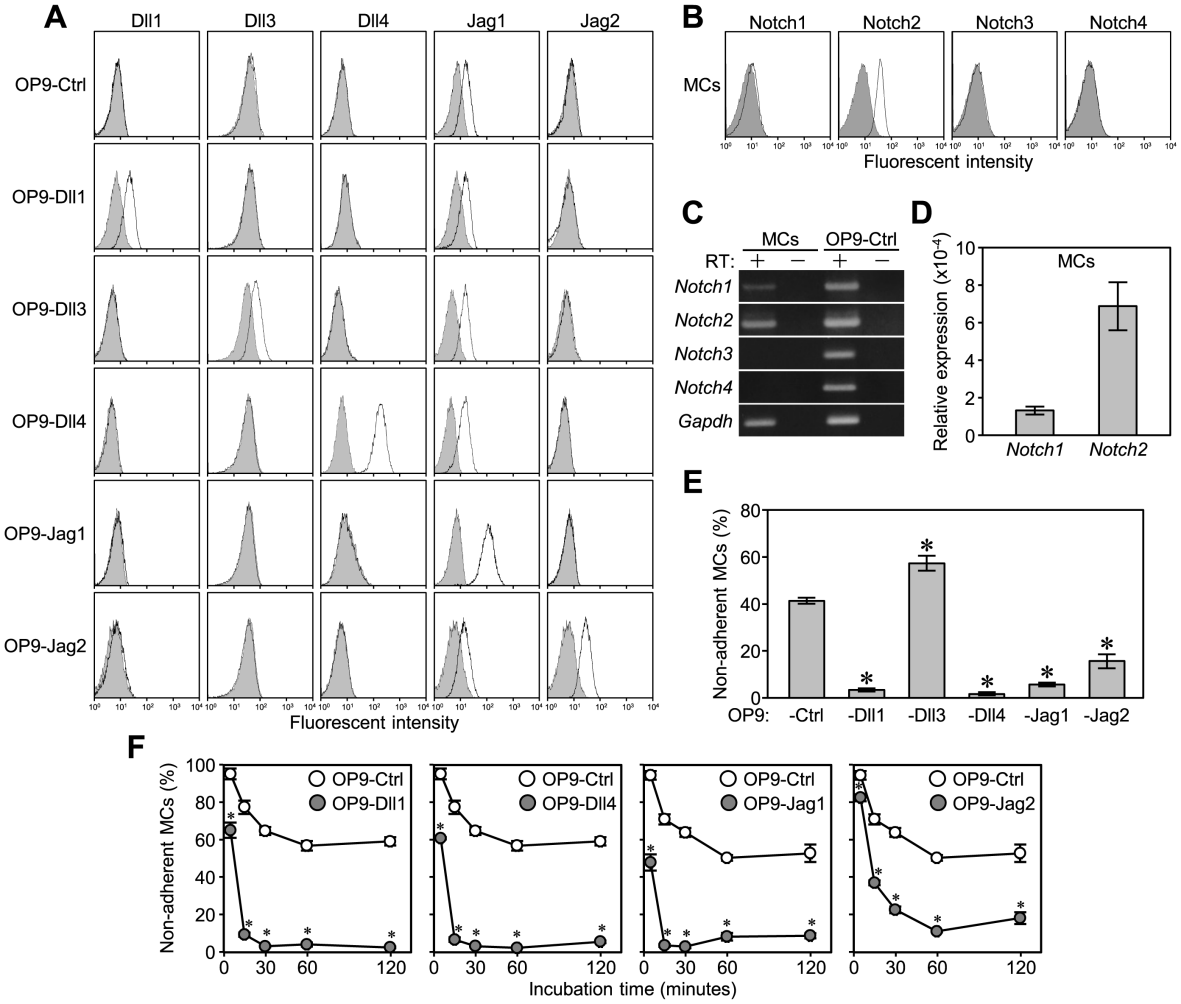
MCs efficiently adhered to OP9-Dll1, -Dll4, -Jag1, and -Jag2, but not OP9-Dll3, than to OP9-Ctrl. (A and B) Flow cytometric analysis of the expression of (A) Dll1, Dll3, Dll4, Jag1, and Jag2 on OP9 stromal cells transduced with each Notch ligand gene, and (B) Notch receptors on MCs after staining with specific mAbs (open histograms) or isotype-matched control mAbs (filled histograms). (C) Total RNA was analyzed by RT-PCR for the expression of Notch receptors in MCs and OP9-Ctrl cells. (D) Relative expression levels of *Notch1* and *Notch2* to *Gapdh* in MCs were analyzed by quantitative RT-PCR. Data represent the mean ± SEM of 3 independent experiments. (E and F) An adhesion assay for MCs on each OP9 cell (E) in a 48-well plate for 60 min and (F) in 96-well plates with serial incubation times of 5, 15, 30, 60, and 120 min. Data represent the percentages of non-adherent MCs (mean ± SEM of triplicate cultures) (*p<0.05 significantly different from OP9-Ctrl at each time point, the Student’s *t-*test).

**Figure 2:**
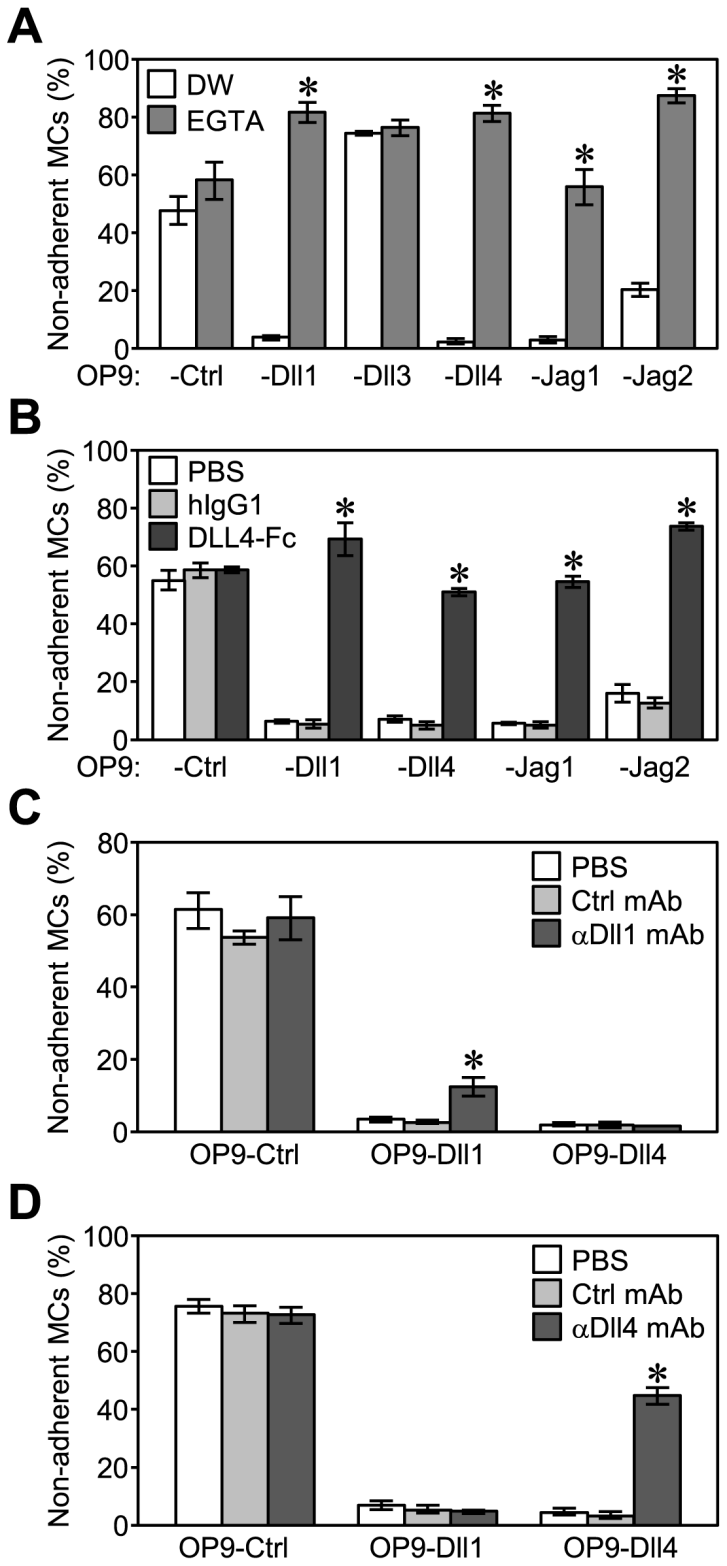
Enhanced MC adhesion to OP9-Dll1, -Dll4, -Jag1, or -Jag2 depended on Notch receptor-ligand interactions. An adhesion assay (60 min) for MCs on each OP9 cell (A) with EGTA (3.0 mM) or the same volume of distilled water (DW, control, 0.6% vol/vol) in a 48-well plate, (B) with 25 µg/ml of soluble recombinant DLL4-Fc, human IgG1 (control), or the same volume of PBS (19.2% vol/vol) in a 96-well plate, (C) with 50 µg/ml of anti-Dll1 mAb, anti-CTLA4 mAb (control), or the same volume of PBS (5.0% vol/vol) in a 96-well plate, (D) with 200 µg/ml of anti-Dll4 mAb, anti-CTLA4 mAb (control), or the same volume of PBS (20.0% vol/vol) in a 96-well plate. Data represent the percentages of non-adherent MCs (mean ± SEM of triplicate cultures) (*p<0.05 significantly different from each control treatment on the same OP9 cells, the Student’s *t-*test).

**Figure 6:**
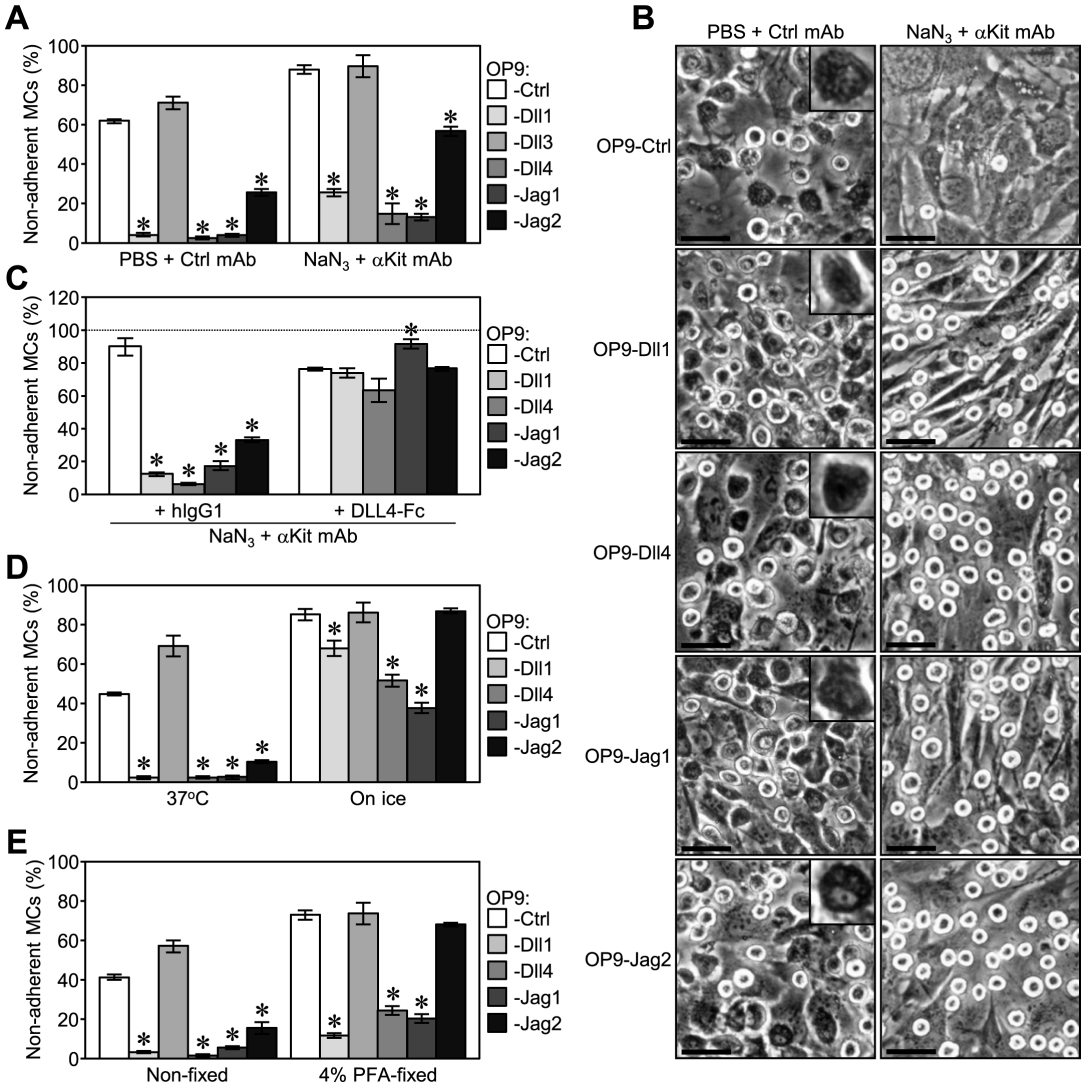
Notch receptor-ligand binding strongly supported the tethering of MCs to stromal cells. (A and B) An adhesion assay with an anti-IL-7Rα mAb (control) or anti-Kit mAb (5 µg/ml each) in the presence of NaN_3_ (50 mM) or the same volume of PBS (0.083% vol/vol) in a 96-well plate. (A) Data represent the percentages of non-adherent MCs (mean ± SEM of triplicate cultures) (*p<0.05 significantly different from OP9-Ctrl with each treatment, the Student’s *t-*test). (B) Representative photomicrographs of adherent MCs on each OP9 stromal cell after the removal of floating cells were shown (original magnification x200). Scale bars; 50 µm. Insets; higher magnification of a spreading adherent MC. (C) An adhesion assay with human IgG1 (control) or DLL4-Fc (25 µg/ml each) in the presence of anti-Kit mAb (5 µg/ml) and NaN_3_ (50 mM) in a 96-well plate. (D and E) An adhesion assay (60 min) in a 48-well plate (D) at 37°C or on ice, and (E) on non-fixed or 4% PFA-fixed stromal cells. (C to E) Data represent the percentages of non-adherent MCs (mean ± SEM of triplicate cultures) (*p<0.05 significantly different from OP9-Ctrl with each treatment, the Student’s *t-*test). In (A), (D) and (E), data displayed significant differences between treatments on the same OP9 cells in most cases (the Student’s *t-*test).
